# Nasopharyngeal carriage of *Streptococcus pneumoniae* serotypes among children in India prior to the introduction of pneumococcal conjugate vaccines: a cross-sectional study

**DOI:** 10.1186/s12879-019-4254-2

**Published:** 2019-07-10

**Authors:** Catherine G. Sutcliffe, Anita Shet, Rosemol Varghese, Balaji Veeraraghavan, Anand Manoharan, Brian Wahl, Sara Chandy, Jack Sternal, Raziuddin Khan, Rakesh Kumar Singh, Mathuram Santosham, Narendra K. Arora

**Affiliations:** 1 Johns Hopkins Bloomberg School of Public Health, 615 N. Wolfe Street, Baltimore, MD 21205 USA; 2 The INCLEN Trust International, F-1/5, 2nd Floor, Okhla Industrial Area Phase – 1, New Delhi, 110020 India; 3 Christian Medical College, Ida Scudder Road, Vellore, Tamil Nadu 632004 India; 4 The CHILDS Trust Medical Research Foundation, 12-A Nageswara Road, Nungambakkam, Chennai, 600034 India

**Keywords:** Pneumococcus, Nasopharyngeal colonization, India, Pediatrics

## Abstract

**Background:**

*Streptococcus pneumoniae* is a major cause of pneumonia, meningitis, and other serious infections among children in India. India introduced the 13-valent pneumococcal conjugate vaccine (PCV) in several states in 2017, and is expected to expand to nationwide coverage in the near future. To establish a baseline for measuring the impact of PCV in India, we assessed overall and serotype-specific nasopharyngeal carriage in two pediatric populations.

**Methods:**

A cross-sectional study was conducted in Palwal District, Haryana, from December 2016 to July 2017, prior to vaccine introduction. Children 2–59 months of age with clinical pneumonia seeking healthcare and those in the community with no clear illness were targeted for enrollment. A nasopharyngeal swab was collected and tested for pneumococcus using conventional culture and sequential multiplex PCR. Isolates were tested for antimicrobial resistance using an E test. Children were considered colonized if pneumococcus was isolated by culture or PCR. The prevalence of pneumococcal and serotype-specific colonization was compared between groups of children using log-binomial regression.

**Results:**

Among 601 children enrolled, 91 had clinical pneumonia and 510 were community children. The proportion colonized with *S. pneumoniae* was 74.7 and 54.5% among children with clinical pneumonia and community children, respectively (adjusted prevalence ratio: 1.38; 95% confidence interval: 1.19, 1.60). The prevalence of PCV13 vaccine-type colonization was similar between children with clinical pneumonia (31.9%) and community children (28.0%; *p* = 0.46). The most common colonizing serotypes were 6A, 6B, 14, 19A, 19F, and 23F, all of which are included in the PCV13 vaccine product. Antimicrobial resistance to at least one drug was similar between isolates from children with clinical pneumonia (66.1%) and community children (61.5%; *p* = 0.49); while resistance to at least two drugs was more common among isolates from children with clinical pneumonia (25.8% vs. 16.4%; *p* = 0.08). Resistance for all drugs was consistently higher for PCV13 vaccine-type serotypes compared to non-vaccine serotypes in both groups.

**Conclusion:**

This study provides baseline information on the prevalence of serotype-specific pneumococcal colonization among children prior to the introduction of PCV in India. Our results suggest a role for pneumococcal vaccines in reducing pneumococcal colonization and antimicrobial resistant isolates circulating in India.

**Electronic supplementary material:**

The online version of this article (10.1186/s12879-019-4254-2) contains supplementary material, which is available to authorized users.

## Background

*Streptococcus pneumoniae* (pneumococcus) is a major cause of pneumonia, meningitis, and other serious infections in children. In 2015, there were an estimated 294,000 deaths in children 1–59 months due to pneumococcus globally [1]. A majority of these deaths occurred in low- and middle-income countries. India accounted for an estimated 68,700 pneumococcal deaths in this age group in 2015 [2]. Increasing antibiotic resistance prevalence threatens to make treatment for pneumococcal disease more challenging and expensive in countries such as India [3].

Pneumococcal nasopharyngeal colonization commonly precedes pneumococcal disease [4]. High nasopharyngeal colonization with pneumococcus has been observed in several studies from India [5–7]. The high prevalence and early acquisition of pneumococcal nasopharyngeal colonization in children in India is likely due to the high prevalence of risk factors for bacterial nasopharyngeal colonization, including household crowding, socioeconomic status, and indoor air pollution [8]. In addition to serving as a reservoir for respiratory infections and invasive diseases, colonization of the nasopharynx is also a source of transmission of pneumococcus to other individuals.

Pneumococcal conjugate vaccine (PCV) has contributed to substantial reductions in pneumococcal disease in children in a variety of epidemiologic settings [9–11]. The direct protection provided by PCV is largely mediated by the ability of the vaccine to reduce acquisition and density of vaccine-type pneumococcal nasopharyngeal colonization [4]. This reduces the spread of vaccine-type pneumococci to others in the community, including those who have not been immunized with PCV, resulting in indirect protection as well.

Given its high prevalence in low- and middle-income countries and its relationship with disease, there is growing interest in using nasopharyngeal colonization with vaccine-type pneumococcus as a surrogate endpoint for monitoring PCV impact. Such data are less expensive to collect and are not prone to the same biases as community-based invasive disease surveillance studies conducted in such settings. Analyses assessing serotypes found in the nasopharynx, however, are complicated by the relationship between colonization duration and serotype invasiveness. Some serotypes commonly found to cause invasive disease are not often isolated from the nasopharynx of healthy children, suggesting these serotypes colonize for a short duration [12, 13]. Nevertheless, methods for estimating the impact of PCV on pneumococcal disease using nasopharyngeal colonization data have been described [14].

India introduced the 13-valent PCV product in 6 states in 2017–19 (Himachal Pradesh, Uttar Pradesh, Bihar, Rajasthan, Madhya Pradesh and Haryana) with plans to extend to additional districts and states beyond 2019. With the aim of establishing a baseline in the pre-PCV period that could be used to measure the impact of PCV in India, we assessed serotype-specific nasopharyngeal carriage in two pediatric populations, namely children with signs and symptoms of pneumonia presenting at healthcare clinics and community-based children without signs of pneumonia or other acute infections, prior to vaccine introduction.

## Methods

### Ethics statement

This study was approved by the institutional review boards at the Johns Hopkins Bloomberg School of Public Health (Baltimore, United States) and the INCLEN Trust International (New Delhi, India).

### Setting, design, and procedures

This study was conducted as part of an ongoing multisite study called Baseline Assessment for *Streptococcus pneumoniae* of India Serotypes (BASIS). The overall objective of the BASIS study was to establish a platform for characterizing the pneumococcal serotypes circulating and causing disease among children in India. The current study was conducted at the SOMAARTH Demographic Development and Environmental Surveillance Site (DDESS) in Palwal District, Haryana [15]. The under-five and infant mortality rates for Haryana are 52 and 42 per 1,000 live births, respectively [16]. The SOMAARTH DDESS is a peri-urban area within Palwal district with a population of approximately 200,000 people living in 51 villages [15]. The population is served by one district-level public hospital and several private healthcare facilities and primary care clinics. Haryana introduced PCV in December 2017.

A cross-sectional study was conducted in the SOMAARTH DDESS from December 2016 to July 2017, prior to vaccine introduction. A convenience sample of two groups of children were targeted for enrollment: children with clinical pneumonia presenting at healthcare clinics and children in the community. The total target enrollment for the study was 600 children; each group was enrolled until the total target was met. Children with clinical pneumonia were recruited from the district hospital, two private hospitals with pediatric clinics and five primary care clinics with a high volume of pediatric patients. On recruitment days, children identified by clinic staff with respiratory symptoms were screened by study staff for enrollment in the study. Children were eligible and considered to have clinical pneumonia if: 1) they were 2–59 months of age; and 2) they had evidence of a fever (either documented at > 38.0 °C or reported); and 3) they had tachypnea, one or more danger signs (altered consciousness, convulsions, poor feeding, bulging fontanelle or stiff neck), or if they were suspected to have sepsis. Children were excluded if there was a strong suspicion of viral infection or their symptoms were consistent with acute bronchiolitis. Children in the community were enrolled from one of the primary care clinics, the immunization clinic at the district hospital, and several anganwadi (daycare) centers (under the Integrated Child Development Scheme). Children were eligible if they were 2–59 months of age and had not experienced symptoms of acute respiratory infection, diarrhea, or rash in the past 48 h. For both groups of children, only one child per family was enrolled. If more than one sibling was present and eligible, then the younger sibling was enrolled.

For all children, written informed consent was obtained from the primary caregiver before enrollment in the study. After enrollment, a questionnaire was administered to the primary caregiver by trained study staff to collect information on demographics, vaccine history (by immunization card or self-report), and medical history (children with clinical pneumonia only). For children with clinical pneumonia, receipt of antibiotics in the past 48 h was assessed through discussion with the caregiver and review of medication brought with them. Based on the information available, the interviewer assessed the likelihood of antibiotic receipt. In addition, a nasopharyngeal swab was collected from all children using a sterile flocked flexible nylon swab (FloQSwab, Copan Diagnostics, Murrieta, CA). Swabs were immediately placed in skim milk-tryptone-glucose-glycerol (STGG) medium, transported on ice to the study laboratory, and stored at -70 °C.

### Laboratory methods

STGG media was prepared at the INCLEN laboratory. Each batch of STGG underwent quality control procedures to ensure that it was sterile and supported the recovery of viable pneumococci.

Nasopharyngeal swabs were shipped to the Central Reference Laboratory at Christian Medical College, Vellore for bacterial culture, antimicrobial susceptibility testing, serotyping and PCR testing. Conventional culture was performed according to the United States Centers for Disease Control and Prevention (US CDC) recommended broth enrichment method [17]. Briefly, 200 μL of the STGG medium was inoculated onto Supplemented Todd Hewitt broth with sterile rabbit serum, incubated for 6 h at 37 °C in a 5–7% CO_2_ incubator, and then plated on to blood agar. After overnight incubation, the plates were screened for alpha hemolytic colonies that were morphologically similar to *S. pneumoniae* and sub-cultured. If more than one morphologically non-identical colony was found, all non-identical colonies were sub-cultured. After confirmation of *S. pneumoniae* using standard protocols [18], isolates were serotyped with the Quellung reaction using pneumococcal antisera from Staten’s Serum Institute (Copenhagen, Denmark) according to standard protocols [18].

All samples were also tested by sequential multiplex (SM) PCR. Briefly, the primary blood agar plate was washed with brain heart infusion broth with 10% glycerol. All colonies present on the plate were harvested. After inactivating DNAse [19], the genomic DNA was purified using the QIAamp DNA mini kit (Qiagen, Hilden, Germany) according to the standardized US CDC protocol [18]. Conventional SM PCR was performed for serotyping of *S. pneumoniae* using the standard protocol [20], with modifications developed by the laboratory [20, 21]. All samples SM PCR positive for *S. pneumoniae* serotypes were also subjected to real time PCR using probe and primers targeting the *lyt*A gene as recommended by US CDC [18].

Antimicrobial susceptibility of isolates identified by conventional culture was tested by establishing the minimum inhibitory concentration value using an E test (BioMérieux, Capronne, France) for penicillin, chloramphenicol, cotrimoxazole, erythromycin, vancomycin and cefotaxime. Interpretation of results as susceptible, intermediate, or resistant was based on the cutoff values defined by the Clinical and Laboratory Standard Institute [22].

### Definitions

For the analysis, a child was considered colonized if *S. pneumoniae* was isolated from the nasopharynx by either culture or SM PCR. Serotype coverage, or the proportion of all isolates that were of a type included in a pneumococcal vaccine product, was calculated for existing vaccines, including PCV10 (1, 4, 5, 6B, 7F, 9 V, 14, 18C, 19F and 23F) and PCV13 (PCV10 plus 3, 6A, 19A), and vaccines in development, including a 10-valent product by a manufacturer in India (1, 5, 6A, 6B, 7F, 9 V, 14, 19A, 19F, 23F) [23], PCV15 (PCV13 plus 22F, 33F) [24], and PCV24 (PCV15 plus 2, 8, 9 N, 10A, 11A, 12F, 15B, 17F, 20) [25]. Cross-protection between serotypes 6A and 6B for PCV10 was assumed based on immunologic [26] and epidemiologic data [26–31]. When SM PCR produced a serogroup (e.g. 6A/B/C/D) instead of a vaccine serotype (e.g. 6B), the serogroup was assumed to be nonvaccine-type (i.e. minimum coverage) for the primary analysis. As a secondary analysis, serogroups were assumed to be vaccine-type (i.e. maximum coverage).

Weight-for-age, length-for-age, and weight-for-length Z-scores were calculated using the World Health Organization child growth standards [32]. Receipt of antibiotics in the past 48 h was defined as ‘no’ if the child had not received any drugs or the child had received drugs and the interviewer assessed antibiotic receipt as ‘unlikely’, was defined as ‘yes’ if the child had received drugs and the interviewer assessed antibiotic receipt as ‘definite’ or ‘likely’, and was defined as ‘unknown’ if receipt of drugs was unknown or the child received drugs but the interviewer could not assess the likelihood of antibiotic receipt.

### Analysis

Data were collected on paper and entered in duplicate into a REDCap database. Discrepancies in data entry were identified and resolved by reviewing the paper forms. The analysis was performed using SAS version 9.4 (SAS Institute Inc., Cary, NC).

Descriptive statistics were used to summarize the characteristics of participants in each group. Comparisons between groups and between those colonized and not colonized were made using chi-square tests for categorical variables and Wilcoxon rank-sum tests for continuous variables. Log-binomial regression was used to compare the overall prevalence of colonization, as well as serotype-specific colonization, between groups (reference group = community children) and adjusted for relevant characteristics found to differ significantly (*p* < 0.05) between groups. As the number colonized with individual serotypes was small, only crude serotype-specific prevalence ratios were calculated.

The performance of culture and SM PCR in detecting *S. pneumoniae* was compared using the Kappa statistic. The sensitivity of each method alone for detecting *S. pneumoniae* was calculated in comparison to using both culture and SM PCR using the capture-recapture method for estimating the total number of colonized children [33].

## Results

Among the 610 children enrolled during the study period, 91 were children with clinical pneumonia and 510 were community children. The characteristics of both groups of children are presented in Table 1.

**Table 1:** Characteristics of children 2–59 months of age in Palwal, India and correlates of pneumococcal colonization

Characteristic	Children with clinical pneumonia (*n* = 91)			Children in the community (*n* = 510)			*p*-value^c^
	Total *N* (%)^a^	Colonized *N* (%)^a^	*p*-value^b^	Total *N* (%)^a^	Colonized N (%)^a^	*p*-value^b^	
Sex			0.75			0.81	0.08
Male	54 (59.3)	41 (75.9)		253 (49.6)	139 (55.1)		
Female	37 (40.7)	27 (73.0)		257 (50.4)	139 (54.1)		
Age (months)			0.98			0.69	0.22
2–11	23 (25.3)	17 (73.9)		130 (25.5)	75 (57.7)		
12–23	27 (29.7)	20 (74.1)		111 (21.8)	60 (54.1)		
24–59	41 (45.1)	31 (75.6)		269 (52.8)	143 (53.2)		
Recruitment location, *n* (%)							< 0.0001
District hospital	15 (16.5)	13 (86.7)	0.21	10 (2.0)	2 (20.0)	0.06	
Private hospitals	6 (6.6)	3 (50.0)		0	0		
Primary care clinics	70 (76.9)	52 (74.3)		14 (2.7)	6 (42.9)		
Anganwadi centers^d^	0	0		486 (95.3)	270 (55.6)		
Weight-for-age Z-score			0.95			0.25	0.31
< −3	22 (24.2)	17 (77.3)		117 (23.0)	64 (54.7)		
−3 to < −2	31 (34.1)	23 (74.2)		138 (27.2)	83 (60.1)		
≥ −2	38 (41.8)	28 (73.7)		253 (49.8)	130 (51.4)		
Length-for-age Z-score			0.60			0.41	0.02
< −3	15 (16.9)	10 (66.7)		110 (21.7)	62 (56.4)		
−3 to < −2	10 (11.2)	7 (70.0)		110 (21.7)	65 (59.1)		
≥ −2	64 (71.9)	50 (78.1)		288 (56.7)	150 (52.1)		
Weight-for-length Z-score			0.33			0.33	0.03
< −3	22 (24.7)	17 (77.3)		96 (18.9)	48 (50.0)		
−3 to < −2	22 (24.7)	14 (63.6)		82 (16.2)	50 (61.0)		
≥ −2	45 (50.6)	36 (80.0)		329 (64.9)	178 (54.1)		
Received antibiotics in past 48 h			0.04		n/a		n/a
No - definitely/unlikely	46 (50.5)	39 (84.8)					
Yes - definitely/likely	18 (19.8)	10 (55.6)					
Unknown	27 (29.7)	19 (70.4)					
Danger signs			1		n/a		n/a
None	71 (80.7)	52 (73.2)					
Any	17 (19.3)	13 (76.5)					
Tachypnea			1		n/a		n/a
No	1 (1.1)	1 (100.0)					
Yes	88 (98.9)	66 (75.0)					
Duration of illness			0.74		n/a		n/a
1–2 days	58 (63.7)	44 (75.9)					
≥ 3 days	33 (36.3)	24 (72.7)					
Hospitalized			0.60		n/a		
No	85 (94.4)	64 (75.3)					
Yes	5 (5.6)	3 (60.0)					

n/a – not applicable

^a^Percentages provided in the ‘Total’ column are column percentages; Percentages provided in the ‘Colonized’ column are row percentages

^b^*p*-value for comparison of the proportion colonized within each study population using chi-square tests

^c^*p*-value for comparison of characteristics between study populations (children with clinical pneumonia and community children) using chi-square tests for categorical variables and Wilcoxon rank-sum tests for continuous variables

^d^Daycare center

### Prevalence and correlates of pneumococcal colonization

**Fig. 1:**
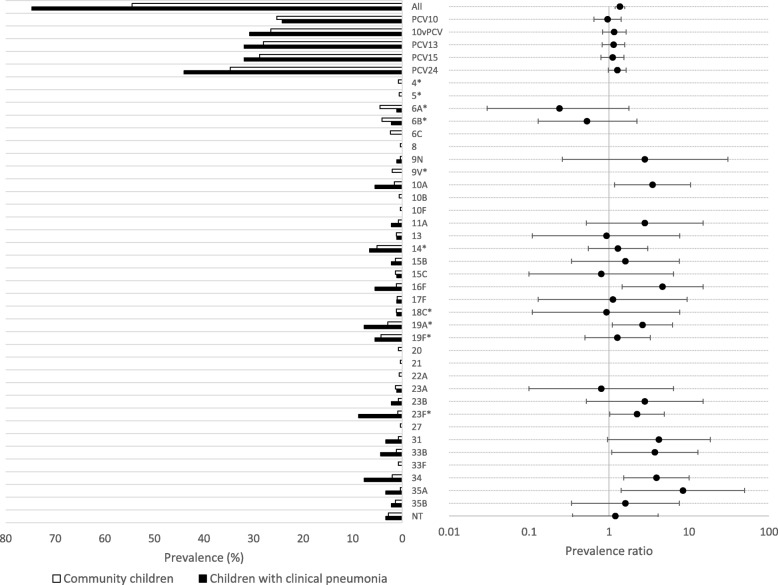
Serotype-specific prevalence of nasopharyngeal colonization among children 2–59 months of age in Palwal, India. Note: * indicates a serotype included in PCV13; NT refers to non-typeable pneumococcus; 10vPCV refers to the 10-valent pneumococcal conjugate vaccine manufactured in India (included serotypes differ from PCV10); Error bars represent 95% confidence intervals

Among children with clinical pneumonia, 74.7% (68/91; 95% confidence interval [CI]: 64.5, 83.3%) were colonized with *S. pneumoniae* (by culture or PCR). Among those colonized, 18 children (26.2%) were colonized with multiple serotypes (16 children with two serotypes and two children with three serotypes; 88 total isolates were identified). Among community children, 54.5% (278/510; 95% CI: 50.1, 58.9%) were colonized with *S. pneumoniae* (see Additional File 1 for the prevalence of colonization by age). Among those colonized, 29 children (26.5%) were colonized with two serotypes (24 children with two serotypes and five children with three serotypes; 312 total isolates were identified). Children with clinical pneumonia were significantly more likely to be colonized with *S. pneumoniae* (Fig. 1; Additional File 2), even after adjusting for age and weight-for-length z-score (adjusted prevalence ratio: 1.38; 95% CI: 1.19, 1.60).

Among children with clinical pneumonia, the prevalence of colonization was significantly lower among those who reported having received antibiotics in the past 48 h (yes: 55.6% vs. no: 84.8% vs. unknown: 70.4%; *p* = 0.04; Table 1). Age, sex, nutrition status, presence of danger signs or tachypnea, duration of illness, and hospitalization were not observed to be associated with colonization (Table 1). Among community children, no characteristics were observed to be associated with colonization.

### Serotype distribution and serotype-specific colonization

While the serotype distributions were different between children with clinical pneumonia (*n* = 88 isolates) and community children (*n* = 312 isolates), there was overlap in common serotypes (see Additional File 3). The six most common serotypes among isolates from children with clinical pneumonia were 23F (8/88; 9.1%), 19A (7/88; 8.0%), 34 (7/88; 8.0%), 14 (6/88; 6.8%), 19F (5/88; 5.7%), and 10A (5/88; 5.7%). The six most common serotypes among isolates from community children were 14 (26/312; 8.3%), 6A (23/312; 7.4%), 19F (22/312; 7.1%), 6B (21/312; 6.7%), 23F (20/312; 6.4%), and 19A (15/312; 4.8%). Serotype coverage for PCV13 was 34.1% (30/88) among children with clinical pneumonia and 48.4% (151/312) among community children. Results for maximum serotype coverage are presented in Additional File 4.

The prevalence of PCV13 vaccine-type colonization among children with clinical pneumonia was 31.9% (29/91) and 28.0% (143/510) among community children (prevalence ratio: 1.14; 95% CI: 0.82, 1.58; Fig. 1 and Additional File 2; see Additional File 4 for maximum colonization prevalence). The prevalence of colonization with serotypes 10A, 16F, 19A, 23F, 33B, 34, and 35A was significantly higher among children with clinical pneumonia compared to community children (Fig. 1 and Additional File 2).

### Comparison of SM PCR and culture for detection of *S. pneumoniae*

The performance of SM PCR was similar to culture in detecting *S. pneumoniae* (Table 2)*,* with good agreement between the two methods (Kappa: 0.72 among children with clinical pneumonia and 0.89 for community children). The sensitivity of culture and SM PCR alone compared to a combination of culture and SM PCR was 89.1 and 93.5%, respectively, among children with clinical pneumonia, and 96.1 and 92.9%, respectively, among community children. Multiple serotypes were more likely to be detected using SM PCR than culture (Table 2). Among children with clinical pneumonia, 15.4% (14/91) of participants had multiple serotypes detected with SM PCR compared to 1.1% (1/91) with culture. An additional four children with clinical pneumonia had multiple serotypes detected using both culture and SM PCR. Among community children, 4.1% (21/510) of participants had multiple serotypes detected with SM PCR compared to 1.4% (7/510) with culture alone. An additional five children had multiple serotypes detected using both culture and SM PCR.

**Table 2:** Comparison of PCR and culture results for detecting *S. pneumoniae* among children in Palwal, India

	Children with suspected pneumonia (*n* = 91)	Children in the community (*n* = 510)
Culture positive, *n* (%)	61 (67.0)	268 (52.6)
PCR positive, *n* (%)	64 (70.3)	259 (50.8)
Culture or PCR positive, *n* (%)	68 (74.7)	278 (54.5)
PCR positive/culture positive, *n* (%)	57 (62.6)	249 (48.8)
PCR positive/culture negative, *n* (%) ^a^	7 (7.7)	10 (2.0)
PCR negative/culture positive, *n* (%) ^b^	4 (4.4)	19 (3.7)
PCR negative/culture negative, *n* (%)	23 (25.3)	232 (45.5)
Multiple serotypes present if culture positive, *n* (%)	1 (1.1)	7 (1.4)
Multiple serotypes present if PCR positive, *n* (%)	14 (15.4)	21 (4.1)
Multiple serotypes present if culture or PCR positive, *n* (%)	18 (19.8)	29 (5.7)

^a^Serotypes: children with clinical pneumonia: 6A/B/C/D, 10A, 19A, 23F, 31, 35F/47F, 39; community children: 5, 7C/B/40, 9 N/L, 10F/C/33C, 14, 16F, 24F/A/B, 25F/A/38, 34, 35B

^b^Serotypes: children with clinical pneumonia: 33B; community children: 10B, 27, 33B, 48, non-typeable

### Antimicrobial resistance

**Fig. 2:**
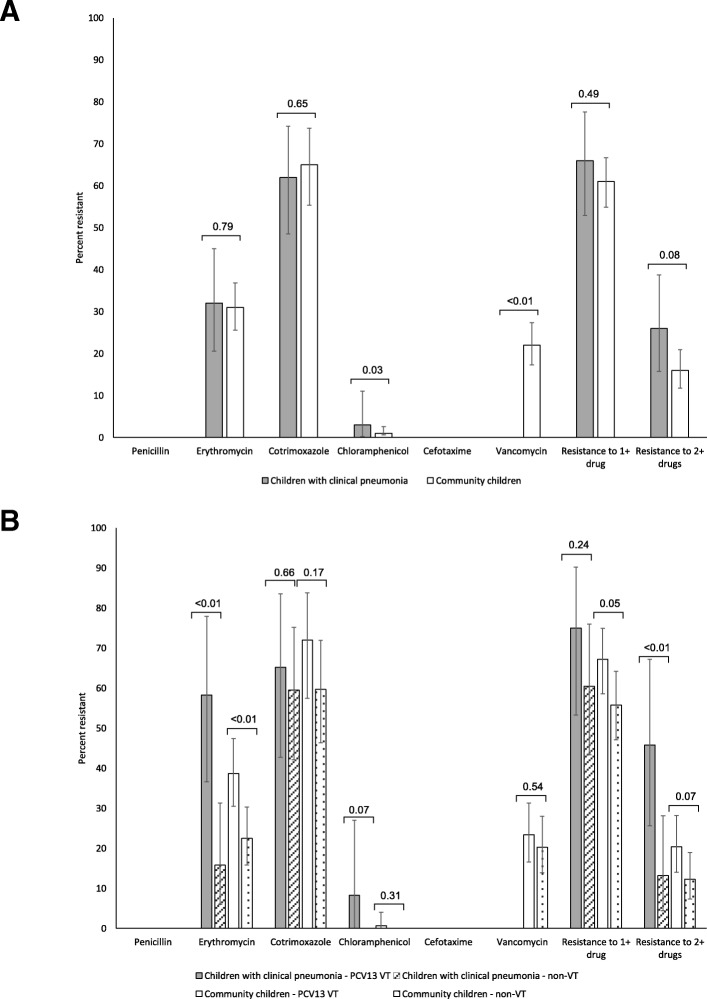
Antimicrobial resistance among pneumococcal isolates in Palwal, India, by group (**a**) and PCV13 vaccine-type (**b**). Note: *P*-values from chi-square tests for comparison between study populations (children with clinical pneumonia and community children) (panel **a**) or between PCV13 vaccine-type (VT) and non-vaccine type (nonVT) serotypes within each study population (panel **b**); Error bars represent 95% confidence intervals; Resistance to cotrimoxazole was evaluated for only 59 and 112 children with clinical pneumonia and community children, respectively

Antimicrobial susceptibility results were available for 62 isolates from children with clinical pneumonia and 272 isolates from community children. Patterns of antimicrobial resistance were similar between isolates from children with clinical pneumonia and community children (Fig. 2a). Resistance to erythromycin (children with clinical pneumonia: 32.3%; community children: 30.6%) and cotrimoxazole (children with clinical pneumonia: 61.7%; community children: 65.2%) were common in both groups of children. Significantly more isolates were resistant to chloramphenicol (3.2% vs. 0.4%; *p* = 0.03) and significantly fewer isolates were resistant to vancomycin (0% vs. 21.8%; *p* < 0.0001) among children with clinical pneumonia compared to community children. Resistance to at least one drug was similar between isolates from children with clinical pneumonia (66.1%) and community children (61.5%; *p* = 0.49). However, more isolates were resistant to at least two drugs (25.8% vs. 16.4%; *p* = 0.08) among children with clinical pneumonia compared to community children, although this result was not statistically significant. Resistance to all drugs was consistently higher for PCV13 vaccine-type serotypes in both groups of children (Fig. 2b). Serotype-specific antimicrobial resistance is presented in Additional File 5.

## Discussion

In this community in Haryana, India, approximately three-quarters of children with clinical pneumonia and half of community children were colonized with *S. pneumoniae,* and approximately a quarter of children in both groups were colonized with PCV13 serotypes. Seven serotypes were significantly more likely to be carried by children with clinical pneumonia compared with community children. Resistance to erythromycin and cotrimoxazole was common among isolates from both groups of children. Resistance was consistently higher among isolates that were PCV13 vaccine serotypes.

Measuring pneumococcal colonization and the distribution of serotypes in the nasopharynx can be used as a way to monitor both the direct and indirect impact of vaccines. Different populations can be targeted for monitoring, including children in the community and children with disease, which were both included in this study. To our knowledge, the current study is the first to describe the prevalence of pneumococcal colonization and distribution of serotypes in children with clinical pneumonia recruited from primary care clinics.

Measuring the prevalence of serotype-specific colonization in the two groups of children allowed us to estimate the serotype-specific disease potential for clinical pneumonia compared to asymptomatic children based in the community. While it cannot be definitively established that colonizing pneumococci played a causal role in the illness among children with clinical pneumonia, this comparison was conducted under the assumption that if the illness was caused by pneumococci, it would likely be of the same serotype as the colonizing pneumococci. We observed that seven serotypes (i.e., 10A, 16F, 19A, 23F, 33B, 34, 35A) were associated with clinical pneumonia without adjusting for possible confounders. A study conducted among children younger than five years in Israel measured the serotype-specific prevalence of pneumococcus in the nasopharynx of radiologically-confirmed pneumonia cases and healthy controls [34]. In that study, serotypes 1, 5, 22F, and 14 had the highest disease potential after adjusting for possible confounders. None of these serotypes was isolated from the nasopharynx of children with clinical pneumonia in the current study, except serotype 14 which was not found to be statistically significantly associated with symptoms in crude analyses. The differences in serotype-specific disease potential between the current study and the study from Israel are likely due to differences in the pneumonia case definitions (i.e., clinical pneumonia versus radiologically confirmed pneumonia) and epidemiological differences between the study settings.

Several studies conducted in India have measured the prevalence of nasopharyngeal colonization with pneumococcus in children younger than five years prior to routine use with PCV. Most studies were conducted in urban areas of South India and enrolled children from hospital outpatient departments or immunization clinics [7, 35–38]. These studies yielded a wide range of estimates, from 6.5% prevalence of colonization among children 3 to 36 months in a hospital-based study in Delhi [36], to 28% among children 3 to 59 months attending an immunization clinic in Bangalore [38]. Studies that have followed children longitudinally, have found a higher peak prevalence of 70% among children 2–6 months in a community-based study in rural South India [6], and 46% among children 1–11 months [5]. The prevalence of colonization will vary across studies depending on many factors, including the age distribution of the study population, enrollment criteria, prevalence of risk factors for colonization, seasonality, and laboratory methods. The high prevalence of colonization observed among community children in this study may be attributable to these factors, as half the study population was younger than two years of age and enrolled from rural and peri-urban area with low socioeconomic status. In addition, colonization in this study was determined based on both culture and PCR, in contrast to the use of only culture in prior studies [5–7, 35–38], which increased the overall prevalence of colonization.

Few studies from India have reported on the distribution of serotypes found colonizing children. Two studies conducted among asymptomatic children in South India found serotypes/groups 6, 14, 15, 19, and 23 to be the most prevalent [6, 39]. While neither of these studies reports on the proportion of isolates covered by PCV13, PCV7 was estimated to account for 53% of carried isolates in one study [39]. The level of serotype coverage observed in this study (i.e., 48% for PCV13 among community children) and other studies from India corresponds with serotype coverage from many developing countries [40]. Similar to the other studies from India, the most common colonizing serotypes in this study were 6A, 6B, 14, 19A, 19F, and 23F, which are all included in the PCV13 product that was recently introduced, suggesting that the vaccine has the potential to impact pneumococcal colonization and disease. Several studies have also reported on the distribution of serotypes among children younger than 5 years of age with invasive disease in India [41–43]. While the common serotypes were similar to those found colonizing children in this study (e.g. 14, 19F, 6A, 6B, 19A, 23F), serotype 1 and 5 were commonly found, which were rare in this study (serotype 5 identified from three community children; serotype 1 not identified from any children). In addition, the proportion of PCV13 vaccine-type serotypes identified from children with invasive disease was higher (74 and 78%) than in this study [41–43].

The use of SM PCR in this study in combination with culture increased not only the overall prevalence of colonization but also the number of serotypes detected. Culture followed by the Quellung reaction is currently accepted as the gold-standard method for pneumococcal serotyping [44, 45], but the test has limited ability to detect multiple serotypes within the same sample. Molecular methods, including real-time PCR, have been found to be more sensitive than culture [46, 47], particularly for detecting low density colonization [48, 49], and are better able to detect colonization with multiple serotypes without the need for bacterial culture [50]. The SM PCR protocol used in this study was developed by the US CDC and uses primers targeting serotype- or serogroup-specific regions in the *cps* loci [51]. The assay allows for the simultaneous detection of capsule specific genes of 40 pneumococcal serotypes, including the most common disease-causing serotypes [52, 53]. However, due to high sequence homology in the pneumococcal capsule region of some serogroups, detection of specific serotypes may be limited in some cases [54]. Given the advantages of molecular methods, future colonization studies should consider their use to better estimate colonization and serotype distributions, particularly after vaccine introduction when colonization density of vaccine serotypes may be reduced.

Resistance to cotrimoxazole and erythromycin were common among both groups of children. This finding is consistent with other pediatric colonization studies from India [35, 38], as well as studies of invasive pneumococcal disease [41, 43], that have found high levels of resistance to cotrimoxazole and potentially increasing resistance to erythromycin. Resistance was higher among PCV13 vaccine-type serotypes, which is consistent with what was observed in countries prior to the introduction of vaccines [55], as these serotypes are successful colonizers that tend to be carried for longer periods of time and are therefore under higher antibiotic pressure. While introduction of PCV leads to declines in vaccine-type serotypes, declines in overall antibiotic resistance may be offset by increasing prevalence and resistance among non-vaccine serotypes [55, 56].

This study had several limitations. First, the sample of children with clinical pneumonia, and thus the number of isolates detected, was relatively small due to the short period of enrollment and the seasonality of respiratory infections in this area. This limited the study’s power to detect correlates of colonization and differences between groups of children. Second, children were enrolled from hospitals and clinics based only on a clinical diagnosis of pneumonia. Chest x-rays were not routinely performed for respiratory infections in this area and infrastructure for performing chest x-rays was not available at the facilities. Therefore, this group may include children who do not have pneumonia and limit the inferences that can be made. Third, colonization with *S. pneumoniae* detected by SM PCR may have been overestimated, due to the possibility of cross-reaction with non-penumococcal species (e.g. *S. mitis*) [57, 58]. However, using both *lyt*A and serotype-specific targets, as well as conventional culture should minimize this concern. Lastly, vaccination status of the children was not available. Collection of vaccine history was attempted, but few children had their immunization card available at the time of the study visit and the vaccine history reported by parents was deemed unreliable. At the time the study was conducted, pneumococcal vaccine was only available in the private sector and uptake was very low due to the cost of the vaccine, therefore it was unlikely that any children received the vaccine. After vaccine introduction, however, it will be important for studies to collect accurate information about receipt of routine and pneumococcal vaccines, and strategies will be needed to improve access to immunization cards.

## Conclusions

In summary, the majority of children with clinical pneumonia and community children were colonized with *S. pneumoniae*. Our data suggest that PCV will have an important impact in India. The most commonly identified colonizing serotypes are all included in the PCV13 product as well as the upcoming 10-valent vaccine product from India. This study provides baseline information on the prevalence of colonization, overall and with vaccine serotypes, prior to the introduction of PCV13 in India. Continued studies in these populations will be needed to monitor the impact of vaccine introduction on vaccine and non-vaccine serotypes and antimicrobial susceptibility.

## Additional files

Additional file 1: Prevalence of pneumococcal colonization by age among children in Palwal, India. The figure depicts the prevalence of pneumococcal colonization by age (in months) among children 2–59 months of age enrolled from the community in Palwal, India from December 2016 to July 2017. Black bars represent the prevalence of colonization with PCV13 serotypes while grey bars represent the prevalence of colonization with non-PCV13 serotypes. (PPTX 58 kb)

Additional file 2: Serotype-specific prevalence of nasopharyngeal colonization among children 2–59 months of age in Palwal, India. The table shows the prevalence of overall, vaccine-type, and serotype-specific pneumococcal colonization by study population. In addition, the crude prevalence ratio and 95% confidence intervals comparing the prevalence of colonization between children with clinical pneumonia to community children are presented. (DOCX 19 kb)

Additional file 3: Distribution of serotypes among children colonized with *Streptococcus pneumoniae* in Palwal, India. The figure depicts the distribution of pneumococcal serotypes found colonizing the nasopharynx by study population. Black bars represent children with clinical pneumonia and white bars represent community children. (PPTX 62 kb)

Additional file 4: Serotype coverage and nasopharyngeal colonization among children 2–59 months of age in Palwal, India. The table shows the serotype coverage and prevalence of colonization with vaccine-type serotypes for different vaccines. Results are presented by study population. For this analysis, when PCR produced a serogroup (e.g. 6A/B/C/D) instead of a vaccine serotype (e.g. 6B), the serogroup was assumed to be vaccine-type, and thus maximum coverage and colonization are presented. (DOCX 17 kb)

Additional file 5: Serotype-specific antimicrobial resistance among pneumococcal isolates in Palwal, India. The table shows levels of serotype-specific antimicrobial resistance among all pneumococcal isolates. Results for both study populations were combined. Only serotypes with results from a minimum of 5 children are reported. (DOCX 22 kb)

## Data Availability

The data that support the findings of this study can be made available from the corresponding author upon reasonable request.

## Abbreviations

DDESS: Demographic Development and Environmental Surveillance Site
PCV: Pneumococcal conjugate vaccine
SM PCR: Sequential multiplex polymerase chain reaction
STGG: Skim milk-tryptone-glucose-glycerol
US CDC: United States Centers for Disease Control and Prevention
