# Supplementary figures and images for: Nasopharyngeal carriage of Streptococcus pneumoniae serotypes among children in India prior to the introduction of pneumococcal conjugate vaccines: a cross-sectional study
Source: BMC Infect Dis. 2019 Jul 10;19:605. doi: 10.1186/s12879-019-4254-2 (PMC6621985; doi:10.1186/s12879-019-4254-2)

## Slide 1
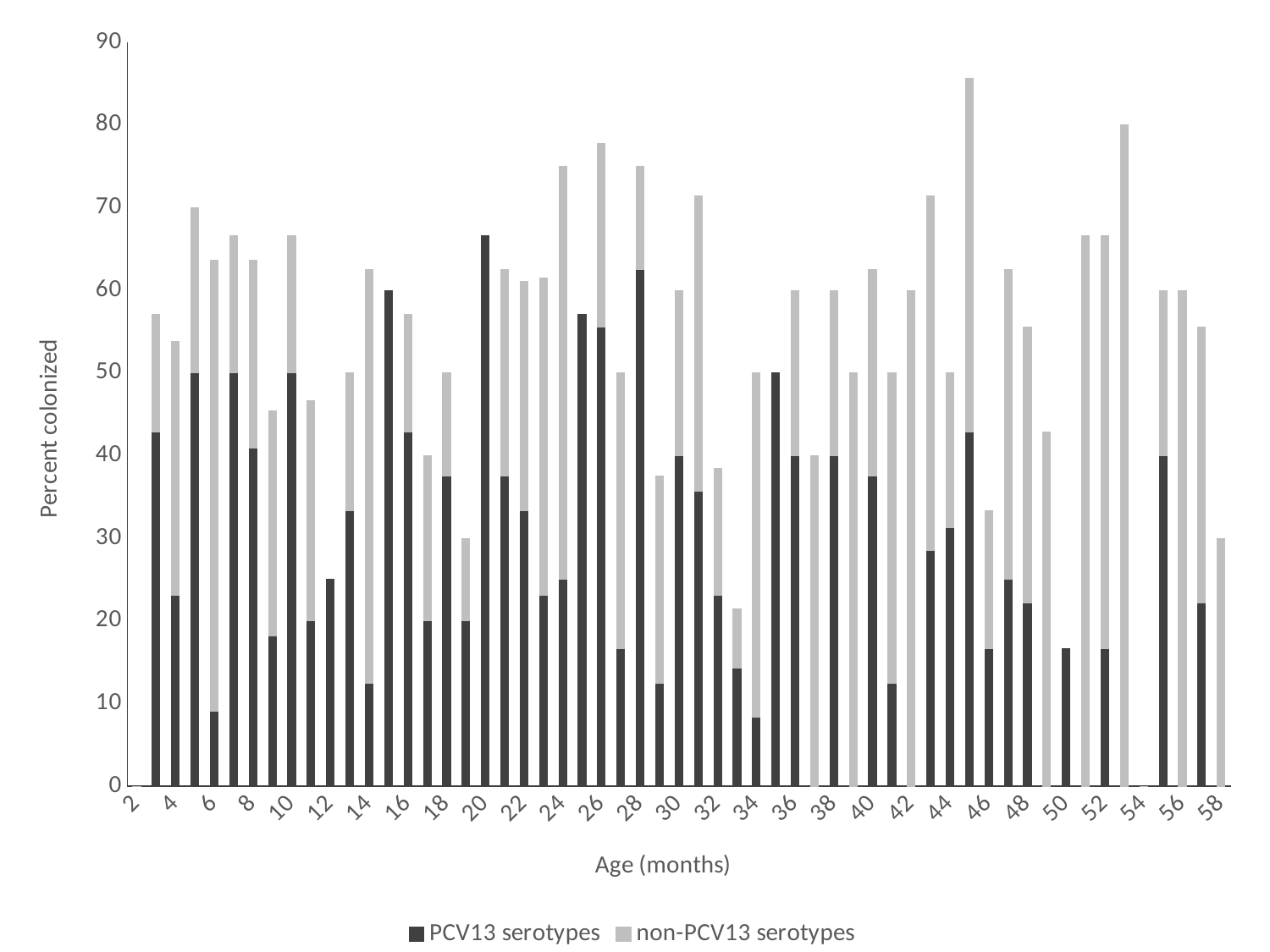

### Chart
| Category | PCV13 serotypes | non-PCV13 serotypes |
|---|---|---|
| 2 | 0.0 | 0.0 |
| 3 | 42.86 | 14.280000000000001 |
| 4 | 23.08 | 30.770000000000003 |
| 5 | 50.0 | 20.0 |
| 6 | 9.09 | 54.55 |
| 7 | 50.0 | 16.67 |
| 8 | 40.91 | 22.730000000000004 |
| 9 | 18.18 | 27.270000000000003 |
| 10 | 50.0 | 16.67 |
| 11 | 20.0 | 26.67 |
| 12 | 25.0 | 0.0 |
| 13 | 33.33 | 16.67 |
| 14 | 12.5 | 50.0 |
| 15 | 60.0 | 0.0 |
| 16 | 42.86 | 14.280000000000001 |
| 17 | 20.0 | 20.0 |
| 18 | 37.5 | 12.5 |
| 19 | 20.0 | 10.0 |
| 20 | 66.67 | 0.0 |
| 21 | 37.5 | 25.0 |
| 22 | 33.33 | 27.78 |
| 23 | 23.08 | 38.46 |
| 24 | 25.0 | 50.0 |
| 25 | 57.14 | 0.0 |
| 26 | 55.56 | 22.22 |
| 27 | 16.67 | 33.33 |
| 28 | 62.5 | 12.5 |
| 29 | 12.5 | 25.0 |
| 30 | 40.0 | 20.0 |
| 31 | 35.71 | 35.720000000000006 |
| 32 | 23.08 | 15.380000000000003 |
| 33 | 14.29 | 7.140000000000001 |
| 34 | 8.33 | 41.67 |
| 35 | 50.0 | 0.0 |
| 36 | 40.0 | 20.0 |
| 37 | 0.0 | 40.0 |
| 38 | 40.0 | 20.0 |
| 39 | 0.0 | 50.0 |
| 40 | 37.5 | 25.0 |
| 41 | 12.5 | 37.5 |
| 42 | 0.0 | 60.0 |
| 43 | 28.57 | 42.86000000000001 |
| 44 | 31.25 | 18.75 |
| 45 | 42.86 | 42.849999999999994 |
| 46 | 16.67 | 16.659999999999997 |
| 47 | 25.0 | 37.5 |
| 48 | 22.22 | 33.34 |
| 49 | 0.0 | 42.86 |
| 50 | 16.67 | 0.0 |
| 51 | 0.0 | 66.67 |
| 52 | 16.67 | 50.0 |
| 53 | 0.0 | 80.0 |
| 54 | 0.0 | 0.0 |
| 55 | 40.0 | 20.0 |
| 56 | 0.0 | 60.0 |
| 57 | 22.22 | 33.34 |
| 58 | 0.0 | 30.0 |

Supplement: Supplementary file 1 — Prevalence of pneumococcal colonization by age among children in Palwal, India. The figure depicts the prevalence of pneumococcal colonization by age (in months) among children 2–59 months of age enrolled from the community in Palwal, India from December 2016 to July 2017. Black bars represent the prevalence of colonization with PCV13 serotypes while grey bars represent the prevalence of colonization with non-PCV13 serotypes. (PPTX 58 kb) [file 12879_2019_4254_MOESM1_ESM.pptx]
