# Supplementary material for: Nasopharyngeal carriage of Streptococcus pneumoniae serotypes among children in India prior to the introduction of pneumococcal conjugate vaccines: a cross-sectional study
Source: BMC Infect Dis. 2019 Jul 10;19:605. doi: 10.1186/s12879-019-4254-2 (PMC6621985; doi:10.1186/s12879-019-4254-2)
Supplement: Supplementary file 2 — Serotype-specific prevalence of nasopharyngeal colonization among children 2–59 months of age in Palwal, India. The table shows the prevalence of overall, vaccine-type, and serotype-specific pneumococcal colonization by study population. In addition, the crude prevalence ratio and 95% confidence intervals comparing the prevalence of colonization between children with clinical pneumonia to community children are presented. (DOCX 19 kb) [file 12879_2019_4254_MOESM2_ESM.docx]

**Additional File 2. Serotype-specific prevalence of nasopharyngeal colonization among children 2-59 months of age in Palwal, India**

| **Serotype** | **Children with clinical pneumonia**  **(n=91) ^a^** | **Community children (n=510) ^a^** | **Crude prevalence ratio**  **(95% confidence interval)** |
| --- | --- | --- | --- |
| All | 68 (74.7) | 278 (54.5) | 1.37 (1.19, 1.58) |
| PCV10 vaccine type ^b^ | 22 (24.2) | 129 (25.3) | 0.96 (0.65, 1.42) |
| 10-valent PCV (India) vaccine type ^b^ | 28 (30.8) | 135 (26.5) | 1.16 (0.83, 1.63) |
| PCV13 vaccine type ^b^ | 29 (31.9) | 143 (28.0) | 1.14 (0.82, 1.58) |
| PCV15 vaccine type ^b^ | 29 (31.9) | 147 (28.8) | 1.11 (0.79, 1.54) |
| PCV24 vaccine type ^b^ | 40 (44.0) | 177 (34.7) | 1.27 (0.98, 1.64) |
| 4* | 0 | 4 (0.8) | --- |
| 5* | 0 | 3 (0.6) | --- |
| 6A* | 1 (1.1) | 23 (4.5) | 0.24 (0.03, 1.78) |
| 6B* | 2 (2.2) | 21 (4.1) | 0.53 (0.13, 2.24) |
| 6C | 0 | 12 (2.4) | --- |
| 8 | 0 | 2 (0.4) | --- |
| 9N | 1 (1.1) | 2 (0.4) | 2.80 (0.26, 30.58) |
| 9V* | 0 | 10 (2.0) | --- |
| 10A | 5 (5.5) | 8 (1.6) | 3.50 (1.17, 10.47) |
| 10B | 0 | 3 (0.6) | --- |
| 10F | 0 | 2 (0.4) | --- |
| 11A | 2 (2.2) | 4 (0.8) | 2.80 (0.52, 15.08) |
| 13 | 1 (1.1) | 6 (1.2) | 0.93 (0.11, 7.67) |
| 14* | 6 (6.6) | 26 (5.1) | 1.29 (0.55, 3.05) |
| 15B | 2 (2.2) | 7 (1.4) | 1.60 (0.34, 7.59) |
| 15C | 1 (1.1) | 7 (1.4) | 0.80 (0.10, 6.43) |
| 16F | 5 (5.5) | 6 (1.2) | 4.67 (1.46, 14.98) |
| 17F | 1 (1.1) | 5 (1.0) | 1.12 (0.13, 9.48) |
| 18C* | 1 (1.1) | 6 (1.2) | 0.93 (0.11, 7.67) |
| 19A* | 7 (7.7) | 15 (2.9) | 2.62 (1.10, 6.24) |
| 19F* | 5 (5.5) | 22 (4.3) | 1.27 (0.50, 3.28) |
| 20 | 0 | 4 (0.8) | --- |
| 21 | 0 | 2 (0.4) | --- |
| 22A | 0 | 3 (0.6) | --- |
| 23A | 1 (1.1) | 7 (1.4) | 0.80 (0.10, 6.43) |
| 23B | 2 (2.2) | 4 (0.8) | 2.80 (0.52, 15.08) |
| 23F* | 8 (8.8) | 20 (3.9) | 2.24 (1.02, 4.93) |
| 27 | 0 | 2 (0.4) | --- |
| 31 | 3 (3.3) | 4 (0.8) | 4.20 (0.96, 18.47) |
| 33B | 4 (4.4) | 6 (1.2) | 3.74 (1.08, 12.98) |
| 33F | 0 | 4 (0.8) | --- |
| 34 | 7 (7.7) | 10 (2.0) | 3.92 (1.53, 10.04) |
| 35A | 3 (3.3) | 2 (0.4) | 8.41 (1.42, 49.61) |
| 35B | 2 (2.2) | 7 (1.4) | 1.60 (0.34, 7.59) |
| Non-typeable | 3 (3.3) | 14 (2.8) | 1.20 (0.35, 4.10) |

* PCV13 vaccine serotype

^a^ n (%) are represented in the column

^b^ Serogroups from PCR assumed to be non-vaccine serotypes
