# Supplementary material for: Nasopharyngeal carriage of Streptococcus pneumoniae serotypes among children in India prior to the introduction of pneumococcal conjugate vaccines: a cross-sectional study
Source: BMC Infect Dis. 2019 Jul 10;19:605. doi: 10.1186/s12879-019-4254-2 (PMC6621985; doi:10.1186/s12879-019-4254-2)
Supplement: Supplementary file 3 — Distribution of serotypes among children colonized with Streptococcus pneumoniae in Palwal, India. The figure depicts the distribution of pneumococcal serotypes found colonizing the nasopharynx by study population. Black bars represent children with clinical pneumonia and white bars represent community children. (PPTX 62 kb) [file 12879_2019_4254_MOESM3_ESM.pptx]

## Slide 1
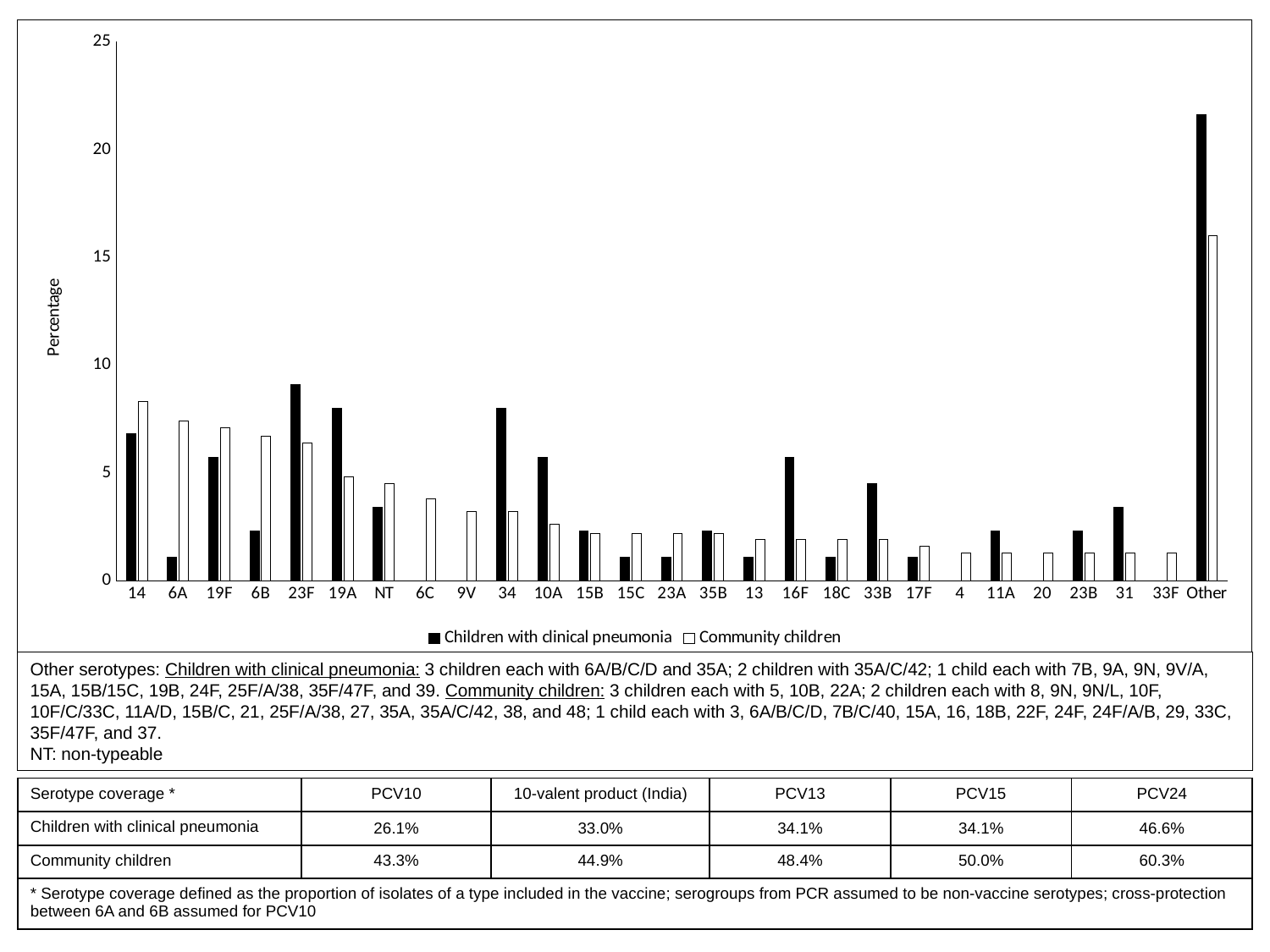

### Chart
| Category | Children with clinical pneumonia | Community children |
|---|---|---|
| 14 | 6.8 | 8.3 |
| 6A | 1.1 | 7.4 |
| 19F | 5.7 | 7.1 |
| 6B | 2.3 | 6.7 |
| 23F | 9.1 | 6.4 |
| 19A | 8.0 | 4.8 |
| NT | 3.4 | 4.5 |
| 6C | 0.0 | 3.8 |
| 9V | 0.0 | 3.2 |
| 34 | 8.0 | 3.2 |
| 10A | 5.7 | 2.6 |
| 15B | 2.3 | 2.2 |
| 15C | 1.1 | 2.2 |
| 23A | 1.1 | 2.2 |
| 35B | 2.3 | 2.2 |
| 13 | 1.1 | 1.9 |
| 16F | 5.7 | 1.9 |
| 18C | 1.1 | 1.9 |
| 33B | 4.5 | 1.9 |
| 17F | 1.1 | 1.6 |
| 4 | 0.0 | 1.3 |
| 11A | 2.3 | 1.3 |
| 20 | 0.0 | 1.3 |
| 23B | 2.3 | 1.3 |
| 31 | 3.4 | 1.3 |
| 33F | 0.0 | 1.3 |
| Other | 21.6 | 16.0 |Other serotypes: Children with clinical pneumonia: 3 children each with 6A/B/C/D and 35A; 2 children with 35A/C/42; 1 child each with 7B, 9A, 9N, 9V/A, 15A, 15B/15C, 19B, 24F, 25F/A/38, 35F/47F, and 39. Community children: 3 children each with 5, 10B, 22A; 2 children each with 8, 9N, 9N/L, 10F, 10F/C/33C, 11A/D, 15B/C, 21, 25F/A/38, 27, 35A, 35A/C/42, 38, and 48; 1 child each with 3, 6A/B/C/D, 7B/C/40, 15A, 16, 18B, 22F, 24F, 24F/A/B, 29, 33C, 35F/47F, and 37.
NT: non-typeable
| Serotype coverage \* | PCV10 | 10-valent product (India) | PCV13 | PCV15 | PCV24 |
| --- | --- | --- | --- | --- | --- |
| Children with clinical pneumonia | 26.1% | 33.0% | 34.1% | 34.1% | 46.6% |
| Community children | 43.3% | 44.9% | 48.4% | 50.0% | 60.3% |
| \* Serotype coverage defined as the proportion of isolates of a type included in the vaccine; serogroups from PCR assumed to be non-vaccine serotypes; cross-protection between 6A and 6B assumed for PCV10 | | | | | |
