# Supplementary material for: Nasopharyngeal carriage of Streptococcus pneumoniae serotypes among children in India prior to the introduction of pneumococcal conjugate vaccines: a cross-sectional study
Source: BMC Infect Dis. 2019 Jul 10;19:605. doi: 10.1186/s12879-019-4254-2 (PMC6621985; doi:10.1186/s12879-019-4254-2)
Supplement: Supplementary file 4 — Serotype coverage and nasopharyngeal colonization among children 2–59 months of age in Palwal, India. The table shows the serotype coverage and prevalence of colonization with vaccine-type serotypes for different vaccines. Results are presented by study population. For this analysis, when PCR produced a serogroup (e.g. 6A/B/C/D) instead of a vaccine serotype (e.g. 6B), the serogroup was assumed to be vaccine-type, and thus maximum coverage and colonization are presented. (DOCX 17 kb) [file 12879_2019_4254_MOESM4_ESM.docx]

**Additional File 4. Serotype coverage and nasopharyngeal colonization among children 2-59 months of age in Palwal, India**

|  | **Serotype coverage ^a,b,c^** | | **Colonization ^b,c^** | |
| --- | --- | --- | --- | --- |
|  | **Isolates from children with clinical pneumonia**  **(n=88)** | **Isolates from community children**  **(n=312)** | **Children with clinical pneumonia**  **(n=91)** | **Community children**  **(n=500)** |
| PCV10 | 27 (30.7) | 136 (43.6) | 24 (26.4) | 130 (25.5) |
| 10-valent PCV (India) | 33 (37.5) | 141 (45.2) | 30 (33.0) | 136 (26.7) |
| PCV13 | 34 (38.6) | 152 (48.7) | 31 (34.1) | 144 (28.2) |
| PCV15 | 34 (38.6) | 157 (50.3) | 31 (34.1) | 148 (29.0) |
| PCV24 | 46 (52.3) | 193 (61.9) | 43 (47.3) | 181 (35.5) |

^a^ Serotype coverage defined as the proportion of isolates of a type included in the vaccine

^b^ serogroups from PCR assumed to be vaccine serotypes; cross-protection between 6A and 6B assumed for PCV10

^c^ n (%) are represented in the column
