# Supplementary material for: Nasopharyngeal carriage of Streptococcus pneumoniae serotypes among children in India prior to the introduction of pneumococcal conjugate vaccines: a cross-sectional study
Source: BMC Infect Dis. 2019 Jul 10;19:605. doi: 10.1186/s12879-019-4254-2 (PMC6621985; doi:10.1186/s12879-019-4254-2)
Supplement: Supplementary file 5 — Serotype-specific antimicrobial resistance among pneumococcal isolates in Palwal, India. The table shows levels of serotype-specific antimicrobial resistance among all pneumococcal isolates. Results for both study populations were combined. Only serotypes with results from a minimum of 5 children are reported. (DOCX 22 kb) [file 12879_2019_4254_MOESM5_ESM.docx]

**Additional File 5. Serotype-specific antimicrobial resistance among pneumococcal isolates in Palwal, India**

| **Serotype ^a^** | **Resistance to each drug**  **n / N (%)** | | | | | | | |
| --- | --- | --- | --- | --- | --- | --- | --- | --- |
|  | **Penicillin** | **Erythromycin** | **Cotrimoxazole** | **Chloramphenicol** | **Cefotaxime** | **Vancomycin** | **Resistance to 1+ drug** | **Resistance to 2+ drugs** |
| 6A | 0 / 24 (0.0) | 10 / 24 (41.7) | 7 / 10 (70.0) | 0 / 24 (0.0) | 0 / 24 (0.0) | 7 / 24 (29.2) | 18 / 24 (75.0) | 5 / 24 (20.8) |
| 6B | 0 / 23 (0.0) | 4 / 23 (17.4) | 4 / 9 (44.4) | 0 / 23 (0.0) | 0 / 23 (0.0) | 4 / 23 (17.4) | 10 / 23 (43.5) | 2 / 23 (8.7) |
| 6C | 0 / 12 (0.0) | 3 / 12 (25.0) | 3 / 4 (75.0) | 0 / 12 (0.0) | 0 / 12 (0.0) | 5 / 12 (41.7) | 8 / 12 (66.7) | 3 / 12 (25.0) |
| 9V | 0 / 10 (0.0) | 1 / 10 (10.0) | 0 / 5 (0.0) | 0 / 10 (0.0) | 0 / 10 (0.0) | 4 / 10 (40.0) | 5 / 10 (50.0) | 0 / 10 (0.0) |
| 10A | 0 / 12 (0.0) | 6 / 12 (50.0) | 3 / 7 (42.9) | 0 / 12 (0.0) | 0 / 12 (0.0) | 0 / 12 (0.0) | 7 / 12 (58.3) | 2 / 12 (16.7) |
| 11A | 0 / 6 (0.0) | 1 / 6 (16.7) | 2 / 2 (100.0) | 0 / 6 (0.0) | 0 / 6 (0.0) | 2 / 6 (33.3) | 5 / 6 (83.3) | 0 / 6 (0.0) |
| 13 | 0 / 7 (0.0) | 3 / 7 (42.9) | 3 / 4 (75.0) | 0 / 7 (0.0) | 0 / 7 (0.0) | 2 / 7 (28.6) | 5 / 7 (71.4) | 3 / 7 (42.9) |
| 14 | 0 / 27 (0.0) | 15 / 27 (55.6) | 10 / 11 (90.9) | 0 / 27 (0.0) | 0 / 27 (0.0) | 5 / 27 (18.5) | 20 / 27 (74.1) | 9 / 27 (33.3) |
| 15B | 0 / 9 (0.0) | 2 / 9 (22.2) | 3 / 5 (60.0) | 0 / 9 (0.0) | 0 / 9 (0.0) | 3 / 9 (33.3) | 6 / 9 (66.7) | 2 / 9 (22.2) |
| 15C | 0 / 8 (0.0) | 3 / 8 (37.5) | 2 / 2 (100.0) | 0 / 8 (0.0) | 0 / 8 (0.0) | 3 / 8 (37.5) | 6 / 8 (75.0) | 1 / 8 (12.5) |
| 16F | 0 / 8 (0.0) | 1 / 8 (12.5) | 5 / 7 (71.4) | 0 / 8 (0.0) | 0 / 8 (0.0) | 1 / 8 (12.5) | 5 / 8 (62.5) | 2 / 8 (25.0) |
| 17F | 0 / 6 (0.0) | 2 / 6 (33.3) | 0 / 3 (0.0) | 0 / 6 (0.0) | 0 / 6 (0.0) | 2 / 6 (33.3) | 3 / 6 (50.0) | 1 / 6 (16.7) |
| 18C | 0 / 7 (0.0) | 2 / 7 (28.6) | 2 / 3 (66.7) | 0 / 7 (0.0) | 0 / 7 (0.0) | 1 / 7 (14.3) | 4 / 7 (57.1) | 1 / 7 (14.3) |
| 19A | 0 / 19 (0.0) | 13 / 19 (68.4) | 9 / 12 (75.0) | 0 / 19 (0.0) | 0 / 19 (0.0) | 3 / 19 (15.8) | 16 / 19 (84.2) | 9 / 19 (47.4) |
| 19F | 0 / 24 (0.0) | 11 / 24 (45.8) | 8 / 9 (88.9) | 1 / 24 (4.2) | 0 / 24 (0.0) | 4 / 24 (16.7) | 18 / 24 (75.0) | 6 / 24 (25.0) |
| 23A | 0 / 8 (0.0) | 0 / 8 (0.0) | 4 / 6 (66.7) | 0 / 8 (0.0) | 0 / 8 (0.0) | 0 / 8 (0.0) | 4 / 8 (50.0) | 0 / 8 (0.0) |
| 23F | 0 / 22 (0.0) | 10 / 22 (45.5) | 9 / 12 (75.0) | 2 / 22 (9.1) | 0 / 22 (0.0) | 3 / 22 (13.6) | 16 / 22 (72.7) | 6 / 22 (27.3) |
| 31 | 0 / 5 (0.0) | 0 / 5 (0.0) | 1 / 3 (33.3) | 0 / 5 (0.0) | 0 / 5 (0.0) | 0 / 5 (0.0) | 1 / 5 (20.0) | 0 / 5 (0.0) |
| 33B | 0 / 10 (0.0) | 2 / 10 (20.0) | 4 / 5 (80.0) | 0 / 10 (0.0) | 0 / 10 (0.0) | 0 / 10 (0.0) | 6 / 10 (60.0) | 0 / 10 (0.0) |
| 34 | 0 / 15 (0.0) | 0 / 15 (0.0) | 3 / 14 (21.4) | 0 / 15 (0.0) | 0 / 15 (0.0) | 0 / 15 (0.0) | 3 / 15 (20.0) | 0 / 15 (0.0) |
| 35A | 0 / 5 (0.0) | 1 / 5 (20.0) | 2 / 3 (66.7) | 0 / 5 (0.0) | 0 / 5 (0.0) | 0 / 5 (0.0) | 2 / 5 (40.0) | 1 / 5 (20.0) |
| 35B | 0 / 6 (0.0) | 2 / 6 (33.3) | 3 / 4 (75.0) | 0 / 6 (0.0) | 0 / 6 (0.0) | 0 / 6 (0.0) | 5 / 6 (83.3) | 0 / 6 (0.0) |
| NT | 0 / 14 (0.0) | 6 / 14 (42.9) | 5 / 7 (71.4) | 0 / 14 (0.0) | 0 / 14 (0.0) | 3 / 14 (21.4) | 10 / 14 (71.4) | 3 / 14 (21.4) |
| p-value ^b^ |  | 0.002 | 0.01 | 0.67 |  | 0.12 | 0.04 | 0.05 |

^a^ Serotypes with results from a minimum of 5 children are reported; serotypes from children with clinical pneumonia and community children are combined

^b^ p-value for comparison of the proportion resistant across serotypes using chi-square tests
